# Chronotropic, Inotropic and Dromotropic Parameters of the Heart and Oxidative Stress in Rats Receiving High Doses of Fructose

**DOI:** 10.31661/gmj.v0i0.1250

**Published:** 2019-05-11

**Authors:** Esmat Radmanesh, Mahin Dianat, Narges Atefipour

**Affiliations:** ^1^Abadan School of Medical Sciences, Abadan, Iran; ^2^Department of Physiology, Persian Gulf Physiology Research Center, Faculty of Medicine, Ahvaz Jundishapur University of Medical Sciences, Ahvaz, Iran

**Keywords:** Electrophysiological Parameters, Oxidative Stress, Rat, Fructose

## Abstract

**Background:**

Many risk factors, including nutritional ones, contribute to cardiovascular diseases (CVDs). Increased fructose consumption, for example, can lead to an increase in CVD risk factors, i.e. an increase in blood lipids and the development of insulin resistance.

**Materials and Methods:**

In the present study, Sprague Dawley rats were divided into two groups: control group (free access to tap drinking water for seven weeks), and a group that received fructose 10% in drinking water for seven weeks, (n═8 per each group). In all groups, before starting the test period and seven weeks after it, electrocardiogram was recorded by Power lab system. Unpaired t-test and two-way ANOVA were used for data analysis. Also, oxidative stress parameters were measured.

**Results:**

In the group received high doses of fructose, a significant reduction (P <0.05) was observed in the PR interval (P<0.001) and a significant increase (P<0.05) in the QTc interval. However, there was no significant change in the RR interval and the voltage of the QRS complex. A significant decrease in catalase, superoxide dismutase and glutathione peroxidase (P<0.05) and a significant increase (P<0.05) in malondialdehyde and lactate dehydrogenase were observed in the group that received fructose in comparison with the control group at the end of the experiment.

**Conclusion:**

According to our results, the chance of arrhythmias in the rats receiving high doses of fructose was possibly due to the increased oxidative stress in the healthy rats.

## Introduction

Cardiovascular diseases (CVDs), by claiming 17.1 million lives a year, bring about a third of universal deaths [1]. One example of these diseases are arrhythmias which are derangement in the normal rhythm of the heartbeat that results from irregularity at transmission or production of impulses in heart or mixture of both [2]. Diabetes, obesity, hypertension, dyslipidemias, cigarette smoking and a sedentary lifestyle are risk factors for cardiovascular diseases. Studies have demonstrated the association between nutritional factors and the incidence of cardiovascular diseases (CVDs) in the world [3]. Fructose is the main part of sweeteners such as honey, high fructose corn syrup (HFCS) and table sugar, and also its intake has quadruplicated since the early 20th century [4]. Augmented fructose consumption can lead to an increase in CVD risk factors, such as an increase in blood lipids [5] and the development of insulin resistance [6]. In cells, imbalance among creation and degradation of reactive oxygen species (ROS) lead to oxidative stress that is a phenomenon connected with pathogenesis mechanisms of numerous cardiovascular diseases including cardiomyopathies, peripheral vascular disease, cerebrovascular disease (stroke), atherosclerosis, coronary heart disease, hypertension, diabetes, inflammatory diseases, heart failure, and cancer [7, 8]. The elevated reactivity of free radicals leads to the creation of bonds between the ROS and other compounds, altering the role and structure of the tissue. ROS can hurt organelles and plasma membranes [9]. The antioxidant enzymes glutathione peroxidase (GPx), superoxide dismutase (SOD) and catalase (CAT) provides the main line of defense in destroying free radicals [8]. Fructose increases oxidative damage by declining antioxidant defenses and intensifying the production of ROS [10-12]. Parto *et al*. have shown that fructose intake leads to pro-inflammatory condition and systemic oxidative stress [13]. According to the results of previous studies, there is a significant relationship between the high doses of fructose and the increase in oxidative stress, and there is also a high oxidative stress in cardiac diseases. Considering that changes in the parameters of the electrocardiogram (ECG) are observed in cardiac diseases, our study was aimed to evaluate the effects of high doses of fructose on ECG parameters, antioxidant enzyme: GPx, CAT, SOD, lactate dehydrogenase (LDH), and production of lipid peroxidation (MDA) in healthy rats.

## Materials and Methods

### Chemicals

MDA, SOD, CAT and GPx kits were purchased from Zell Bio GmbH Co. (Germany). Xylazine (2%) and ketamine HCl (10%) were obtained from Alfasan Co. (Netherlands). LDH kit was purchased from Parsazmun (Iran).

### Animals and Treatments

Sprague-Dawley rats were divided into two groups: the control group received free access to tap drinking water for seven weeks and the group received fructose 10% in drinking water for seven weeks, (n ═ 8 in each group). Before and seven weeks after starting the experiment period, ECG was recorded by Power lab system in all groups. The rats were kept at temperature 22 ± 2 °C, 50% humidity, 12-hour dark-light cycle and had free access to tap water and standard rat chow diet (Pars Co., Iran). They were treated according to the guidelines of the animal care and use of the Ethics Committee of Abadan School of Medical Sciences, Abadan, Iran (code: IR.ABADANUMS.REC.1395.86).

### ECG Recording

The ECG was recorded before starting the test period and seven weeks after it, by Power lab system (AD-Instruments, Australia).

Fifteen minutes after induction of anesthesia, limb lead II electrocardiogram was recorded to find out voltage of QRS (to evaluate inotropic effect), P-R interval and QT interval (to evaluate dromotropic effect), and heart rate and RR interval (to evaluate chronotropic effect). The formula to correct QT (QTc) is bazzet’s formula: (QTc [QT corrected for HR] = QT/square root RR [RR interval: 60/heart rate]).

### Measurement of LDH, MDA, and Antioxidant Enzymes

Seven weeks after the start of the test period, blood sample accumulated from the right atrium of the heart in the anesthetized rats was transported into EDTA containing tubes. After centrifuging at 4000 g for 10 min, the plasma was reserved at -80 °C for biochemical analysis. LDH, antioxidant enzymes (SOD, CAT, and GPx) and MDA were measured by commercial kits

### Statistical Analysis

Data were analyzed by GraphPad Prism6 and expressed as mean ± SEM. Comparisons between groups were performed using unpaired t-test. Two-way ANOVA was used for multiple comparison tests, followed by LSD. P-value <0.05 was considered statistically significant.

## Results

### Effect of High Doses of Fructose OnChronotropic, Inotropic and Dromotropic Parameters

**Figure 1:**
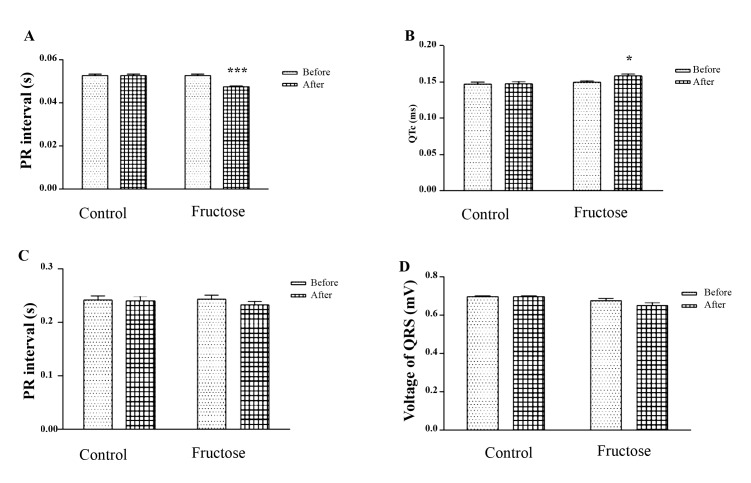
Effect of high doses of fructose on electrophysiological properties of the heart A: PR interval, B: QTc interval, C: RR interval and D: Voltage of QRS. Results are expressed as mean ± SEM of 8 samples per group. Two- way ANOVA was used followed by the LSD test. *P<0.05, ***P<0.001 vs. Control group.

A comparison of PR interval, QTc (dromotropic parameters), RR interval (chronotropic parameters) and voltage of QRS (inotropic parameters**)** did not confirm a significant change on the first day of research. There was a significant decrease in PR interval (P<0.001) and a significant increase in QTc (P<0.05) of the fructose group in comparison with the control group on the last day of research. However, in the RR interval and voltage of QRS, no significant change was observed (Figure-1).

### Effect of High Doses of Fructose On Antioxidant Enzyme

**Figure 2:**
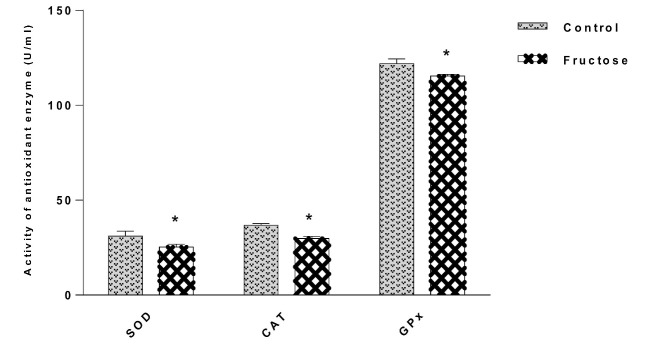
Effect of high doses of fructose on the activity of the antioxidant enzyme (SOD, CAT, and GPx). Results are expressed as mean ± SEM of 8 samples per group. Two- way ANOVA was used followed by the LSD test. *P<0.05 vs. Control group.

A significant decrease in CAT, GPx, and SOD (P<0.05) occurred in the group that received fructose in comparison with the control group on the last day (Figure-2).

#### Effect of High Doses of Fructose On Production of MDA

**Figure 3:**
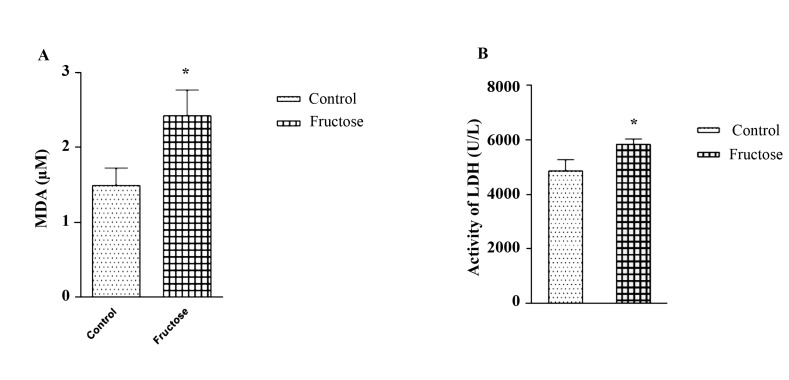
Effect of high doses of fructose on the activity of MDA (A) and LDH (B). Results are expressed as mean ± SEM of 8 samples per group. Unpaired t-test was used. *P<0.05 vs. Control group.

MDA showed a significant increase (P<0.05) in the fructose group in comparison with the control group on the last day (Figure-3A).

### Effect of High Doses of Fructose On Production of LDH

LDH showed a significant increase (P<0.05) in the fructose group in comparison with the control group on last day (Figure-3B).

## Discussion

The aim of our study was to evaluate the effects of high doses of fructose on ECG parameters, an antioxidant enzyme, cardiac marker (LDH) and a marker of lipid peroxidation (MDA) in normal rats. In our study, we observed a significant increase in QTc and a significant decrease in PR interval in fructose group, but in RR interval and voltage of QRS, no significant changes were found. Utilization of sugar-sweetened beverages has been shown to be associated with the development of cardiovascular diseases [14]. Concentrations of low-density lipoprotein (LDL) cholesterol oxidized LDL, small dense LDL, apolipoprotein B increased significantly due to consuming fructose-sweetened beverages [15] and these changes may be related to the augmented risk of CVD [16]. In our previous study, it was shown that high fructose consumption increases the TG, LDL and VLDL levels of plasma in fructose-induced hypertension rats [17]. The present study was aimed to determine the effects of high doses of fructose on the antioxidant enzyme, cardiac marker (LDH) and marker of lipid peroxidation (MDA) in normal rats. In the present study, LDH, a tissue marker for cardiac disorders, in all animal tissues, increased significantly in the group receiving high doses of fructose. In this study, MDA as a marker of lipid peroxidation in the group high doses of fructose increased significantly while the amount of CAT, SOD, and GPx as antioxidant enzyme decreased significantly. In a study by Thirunavukkarasu and Anuradha, in fructose-fed rats group, they observed not only a significant increase in the levels of thiobarbituric acid-reactive substances, diene conjugates, and lipid peroxides but also a decrease in the antioxidant system in high-dose-fructose-fed rats group. They also reported an increase in insulin resistance, free fatty acids, glucose, insulin, and triglycerides in fructose-fed rats [12]. In another study, the antioxidant capacity represented by ascorbic acid, glutathione peroxidase and copper/zinc superoxide dismutase activities were found to be lower in the blood of fructose-fed group. GPX mRNA expression reduced in livers of hypertensive rats fed with the fructose diet [10]. In fructose-fed rats group impaired insulin sensitivity, hypertriglyceridemia, hyperglycemia, hyperinsulinemia, increase in values of protein carbonyl groups (44%), lipid peroxidation (91.3%), a decrease in activities of enzymatic antioxidants, and GSH levels (42.1%) were observed [13]. Acute fructose (5 μmol/g) (fructose group) administration augmented levels of carbonyl content and TBA-RS, indicating lipid peroxidation and protein damage. Furthermore, while there was a decrease in CAT activity, SOD activity increased. The levels of GSH, nitrate, and nitrite were not altered by acute fructose administration. The data indicate that fructose stimulates oxidative stress in the cerebral cortex, resulting in changes in SOD and CAT activities and inducing lipids, proteins, and oxidation [18]. Chronic fructose feeding may lead to hypertension in mice [19]. Left ventricular hypertrophy was induced in high fructose diets in rats, which seems to be associated with stimulation of sympathetic activity [20]. In Axelsen *et al*., the high-fructose consumption resulted in significantly enlarged hearts. In isolated perfused hearts, the baseline values for ±dP/d*t* were higher in the high-fructose group compared with controls, and heart rates were lower in the high-fructose group compared with control group in the isolated hearts, but higher in in vivo study, suggestive of an increased sympathetic tone in the high-fructose group [21]. In Ghosian Moghadam *et al*., the effects of diabetes-induced oxidative stress and the role of lipid peroxidation was demonstrated [22]. Our results provide evidence that high doses of fructose can increase QTc interval, MDA, and LDH but decrease antioxidant enzyme and PR interval in normal rats; however, no significant change in RR interval and voltage of QRS was observed.

## Conclusion

The results of our study showed that the high consumption of fructose has harmful effects on the heart and can be due to its effect on increasing oxidative stress. The high doses of fructose with decreased PR and increased QTc can increase the likelihood of arrhythmia in healthy rats.

## Acknowledgment

Authors gratefully acknowledge the help and financial support of the Abadan School of Medical Sciences, Abadan, Iran, with grant number: IR.ABADANUMS.REC.1395.86.

## Conflict of Interest

The authors declare no conflicts of interest.
